# Poly(3-hydroxybutyrate) Nanocomposites with Cellulose Nanocrystals

**DOI:** 10.3390/polym14101974

**Published:** 2022-05-12

**Authors:** Catalina Diana Usurelu, Stefania Badila, Adriana Nicoleta Frone, Denis Mihaela Panaitescu

**Affiliations:** National Institute for Research and Development in Chemistry and Petrochemistry—ICECHIM, 202 Splaiul Independentei, 060021 Bucharest, Romania; usurelu_catalina@yahoo.ro (C.D.U.); stefania.badila@yahoo.com (S.B.)

**Keywords:** nanocomposites, polyhydroxyalkanoates, cellulose nanocrystals

## Abstract

Poly(3-hydroxybutyrate) (PHB) is one of the most promising substitutes for the petroleum-based polymers used in the packaging and biomedical fields due to its biodegradability, biocompatibility, good stiffness, and strength, along with its good gas-barrier properties. One route to overcome some of the PHB’s weaknesses, such as its slow crystallization, brittleness, modest thermal stability, and low melt strength is the addition of cellulose nanocrystals (CNCs) and the production of PHB/CNCs nanocomposites. Choosing the adequate processing technology for the fabrication of the PHB/CNCs nanocomposites and a suitable surface treatment for the CNCs are key factors in obtaining a good interfacial adhesion, superior thermal stability, and mechanical performances for the resulting nanocomposites. The information provided in this review related to the preparation routes, thermal, mechanical, and barrier properties of the PHB/CNCs nanocomposites may represent a starting point in finding new strategies to reduce the manufacturing costs or to design better technological solutions for the production of these materials at industrial scale. It is outlined in this review that the use of low-value biomass resources in the obtaining of both PHB and CNCs might be a safe track for a circular and bio-based economy. Undoubtedly, the PHB/CNCs nanocomposites will be an important part of a greener future in terms of successful replacement of the conventional plastic materials in many engineering and biomedical applications.

## 1. Introduction

The unprecedented rhythm of using plastics derived from fossil fuels has led to unmanageable environmental problems. These issues are significantly more serious in the packaging industry, where the continuous increase in the out-of-home food consumption has led to an enormous growth in the production and use of packaging materials. Moreover, the COVID-19 pandemic has accelerated the disposal of fossil-fuel-derived plastics from food packaging and medical fields [1,2]. Being the largest and growing consumer of plastics, the packaging industry represents the primary supplier of waste plastics from the environment [1,2,3]. All this mismanaged plastic waste is becoming a source of soil and groundwater pollution that eventually enters our food chain as microplastics [4]. Once inside the human body, the microplastics can cause toxicity, oxidative stress, cytokine secretion, cell damage, and inflammatory and immune reactions [4]. As a result, continuous research efforts are carried out worldwide for the development of more sustainable and environmentally friendly packaging materials derived from renewable feedstocks, including agro, microbial sources, and biomasses [3]. In this context, biopolymers synthesized via bacterial fermentation, like the ones from the polyhydroxyalkanoates (PHAs) family that includes poly(3-hydroxybutyrate) (PHB) and poly(3-hydroxybutyrate-co-3-hydroxyvalerate) (PHBV) polymers, are considered promising candidates for replacing the fossil fuel-based polymeric materials and addressing the waste disposal issue [5,6]. These biopolymers exhibit several features similar to those of fossil fuel-based polymers and, additionally, can be easily degraded by the action of enzymes and living organisms, eliminating the need for disposal systems [7]. Most importantly, their degradation products possess no danger to human beings or the environment. Moreover, being biocompatible, PHB is also suitable for use in a variety of medical applications [7,8]. 

PHB is by far the most intensively studied biopolymer from PHAs’ family and its global market is predicted to reach $221.14 million by 2027 [9]. PHB exhibits remarkable properties such as optical activity, stiffness, and high oxygen barrier properties [8]. However, despite these desirable properties its widespread industrial application, especially in the food packaging sector, has been limited due to its inherent physical aging, low ductility, limited processability, and low crystallization rates in the absence of nucleating agents [7,8]. Multiple strategies to overcome PHB’s drawbacks and to enhance its performance for targeted applications were proposed over time, including blending with other bio-based polymers [10], graft or block copolymerization, the use of specific additives (plasticizers, stabilizers, chain extenders, nucleating agents etc.), and the addition of natural micro-/nano- reinforcing agents [7,8]. 

PHB-based polymer nanocomposites, which are obtained by the incorporation of nanosized particles into the polymer matrix, exhibited clear improvement of properties compared to neat PHB, especially with respect to mechanical and barrier performances [11]. Owing to their outstanding properties, such as renewability, biodegradability, good thermal resistance, low density, high specific strength and stiffness, nontoxicity and lack of corrosion, cellulosic nano-reinforcements are some of the most suitable fillers for PHB [12,13]. In addition, they may contribute to preserving the properties of food products due to their barrier properties and ability to carry antioxidant and antibacterial agents. Different cellulose nanofillers, nanocrystals or nanofibers, were used to overcome some of PHB’s weaknesses, such as its slow crystallization, brittleness, modest thermal stability, and low melt strength [14,15,16,17,18]. However, the strong hydrophilicity of cellulose nanofillers prevents their good dispersion in the hydrophobic PHB matrix and reduces their reinforcing ability. Therefore, surface treatment by TEMPO oxidation or plasma treatment [14], grafting with silanes [18,19], and polymers compatible with PHB [18] were tested as methods to improve the adhesion between the cellulose nanofillers and the PHB or PHBV matrix. 

One route to improve the properties of PHB is the addition of cellulose nanocrystals (CNCs) as nano-reinforcements and the fabrication of PHB/CNCs nanocomposites. In this contribution we provide a comprehensive review on the PHB/CNCs nanocomposites intended for packaging application. Particular attention was paid to choosing the adequate processing technology for producing the PHB/CNCs nanocomposites and an appropriate surface treatment for the CNCs as key elements for achieving a good interfacial adhesion, superior thermal stability, and mechanical performances for the resulting formulations. Although there are several general reviews on biocomposites with cellulose nano-reinforcements, no review dealing with the PHB/CNCs nanocomposites was published so far. The information provided in this review may be considered as a basis for finding new strategies to reduce the manufacturing costs or to design better technological solutions for the large-scale production of PHB/CNCs nanocomposites. 

## 2. Poly(3-hydroxybutyrate)

Poly(3-hydroxybutyrate) (PHB) is the most well-known and widely used member of PHAs [20], a family of biodegradable polyesters derived from the microbial fermentation of different carbon sources [21]. Although chemical synthesis is also possible [22], the main method of producing PHB is its extraction from various bacterial strains which are capable of synthesizing and accumulating PHB intracellularly, as carbon and energy reserve, under nutrient limiting conditions [23]. Due to its biodegradability in both soil and marine environments [24], non-toxicity, biocompatibility, and a melting temperature, elastic modulus and tensile strength similar to that of isotactic polypropylene [25], PHB has drawn increasing attention as an environmentally friendly substitute for petroleum-based thermoplastics, such as polypropylene (PP) and polyethylene (PE) [26]. The world’s rising concern on plastic pollution and oil resources depletion supported the study of PHB—based materials for potential applications in packaging (films, bags, bottles, containers etc.) [27], biomedicine (surgical sutures, drug delivery systems, surgical meshes, wound dressings, scaffolds for tissue engineering etc.) [28] and agriculture (carriers for the slow release of pesticides, herbicides, fertilizers etc.) [29]. However, the high degree of crystallinity of PHB [30] that imparts brittleness [14], the thermal degradation at temperatures just above its melting temperature [31] that narrows its processing window [26], the low elongation at break [32] and the high production costs [14], are serious disadvantages which have restrained the use of PHB on a large scale [33]. 

In recent years, efforts have been made to eliminate these disadvantages. For example, in order to reduce the production costs of PHB, the replacement of the noble carbon sources such as glucose, mannose, and lactose [34] with low-cost agro-industrial byproducts and residues has been proposed [35]. Waste glycerol from biodiesel fuel production [36], corn waste [37], wheat straw [38], rice straw [39], dairy waste [40], sugarcane vinasse, and molasses [41] are just a few of the industrial and agricultural by-products and residues that were successfully employed as carbon sources in the obtaining of PHB by microbial fermentation [42]. Regarding the improvement of the mechanical properties and thermal stability of PHB, several methods have been developed. One method consists in the incorporation of secondary flexible monomers such as 3-hydroxyvalerate, 4-hydroxybutyrate, or 3-hydroxyhexanoate [43] in the main chain of PHB that led to the formation of PHBV [44], poly (3-hydroxybutyrate-co-4-hydroxybutyrate) (P4HB) [45], or poly(3-hydroxybutyrate-co-3-hydroxyhexanoate) (PHBHH) [44] copolymers. Despite the fact that these PHB copolymers have shown higher flexibility, lower melting temperatures and wider processing windows as compared to the pristine PHB [46], they still have poor mechanical properties or are difficult to be obtained through efficient synthesis processes, which has limited their utilization for commercial use [47]. A second strategy involves the use of petroleum-based (dioctyl adipate, dioctyl phthalate, dibutyl phthalate, polyethylene glycol, polyadipates etc.) or bio-based (glycerol, glycerol triacetate, triethyl citrate, soybean oil, epoxidized soybean oil etc.) [48] plasticizers, which are known to increase the flexibility of PHB and to decrease its glass transition temperature (T_g_). However, plasticizers may degrade at temperatures lower than PHB, accelerating its thermal degradation during melt processing, or migrate at the surface of the material, altering its mechanical properties [49]. A third method implies the melt blending of PHB with polymers such as medium chain length-PHAs [10], poly(caprolactone) (PCL), polyethylene glycol (PEG), poly(butylene adipate-co-terephthalate) (PBAT), and poly(butylene succinate) (PBS) etc. [10,50,51]. In this case, it has been shown that the obtained blends have improved flexibility, toughness, and processability as compared to the pure PHB. However, in many situations the improvements were not at the anticipated level due to the poor miscibility between PHB and the second polymer in the melt state [52]. Another method involves the addition to PHB of various nanofillers such as titanium dioxide (TiO_2_) [53], zinc oxide (ZnO) [54], carbon nanotubes (CNTs) [55], clays [56], and nanocellulose [16,17]. Using these nanofillers makes possible the obtaining of PHB-based nanocomposites with superior thermal stability and increased mechanical and barrier properties as compared to the neat PHB [53,54,55,56]. Among them, special attention has been paid to nanocellulose fillers due to their renewability, biodegradability, and superior mechanical properties. 

## 3. Cellulose Nanocrystals

Cellulose is the nature’s most abundant biopolymer and can be extracted from various sources such as plants, marine life, fungi, and bacteria [57]. Regardless of its source, the prime structural unit of cellulose is comprised of linear chains of D-glucose linked by repeating β-1,4-glycosidic bonds, followed by a 180° rotation for the next linkage [57,58]. Owing to its large network of intermolecular and intramolecular hydrogen bonds, cellulose is insoluble in water and most organic solvents. The degree of polymerization and molecular weight of cellulose are governed by both the cellulosic source and the methods employed to produce it [59]. Cellulose with nano-scale structural dimensions, referred to as nanocellulose, possesses high surface area, unique morphology, specific high strength and modulus, renewability, customizable surface chemistry, and good biocompatibility, offering myriad of opportunities for medical and engineering applications. As filler in polymers, nanocellulose has the advantage of being a stable material that cannot be melted during melt processing due to the high level of hydrogen bonding. Thus, nanocellulose can be used as a reinforcing agent for biopolymers, especially in the field of industrial packaging, where the melt processing techniques specific to thermoplastic polymers are intensively used [59].

Depending on the preparation methods and sources, nanocellulose can be classified into three categories, namely cellulose nanocrystals (CNCs), cellulose nanofibers (CNFs), and bacterial nanocellulose (BC). CNCs are one of the most important nanocelluloses that are produced at an industrial level using chemical treatments. Besides the wood and lignocellulosic fibers, a more convenient alternative from both an environmental and an economic point of view is represented by the agriculture along with food and beverage-processing waste and byproducts. Thus, CNCs are predominantly extracted from corn cobs [60], tea stalk [61], soy hulls [62], apple [63] and grape pomace [64], pineapple leaves [65], soybean [66], plum shells [67], barley [68] and garlic [69] straws, tomato peel waste [70], sugarcane bagasse [71], orange peel waste [72], or chili leftovers [73]. However, the exploitation of municipal waste and papermaking sludge may represent other alternative sources [58,74]. 

Different strong (sulfuric, hydrochloric and hydrobromic acids) and weak (phosphoric, citric and formic acids) acids were used for breaking the glycoside bonds in cellulose [75]. Acid hydrolysis involves the hydrolytic cleavage of the amorphous regions from the cellulose fibers, when the crystalline region domains are left behind. Reaction temperature and duration as well as the acid type and its concentration are the main parameters that determine the size and morphology of the isolated CNCs [76]. Thus, CNCs possess whisker or a short-rod-like morphology with uniform sizes ranging from 100 to 200 nm in length and 10 to 30 nm in diameter [77,78]. The use of weak acids leads to cellulose fibers with low crystallinity and fibrous morphology as a result of the low dissociation constant of these acids [76]. Despite being a simple and fast isolation method, sulfuric acid hydrolysis has the advantage of yielding CNCs with higher crystallinity degree (over 90%) and also leads to the sulfate esterification of the CNCs surface, which enhances CNCs’ phase stability in aqueous medium [79]. However, sulfate esterification decreases their thermal stability in the case of thermal treatments. Moreover, CNCs’ almost perfect crystalline structure ensures high mechanical properties such as tensile modulus and tensile strength, which are crucial for further applications [80]. It is worth mentioning that when incorporated in the polymer matrices, CNCs develop a network-like formation, upgrading the polymer’s gas barrier and migration properties to a greater extent than the nanoclays or carbon-based materials [8]. However, the strong hydrophilic property of CNCs raises compatibility and dispersibility issues when combined with other polymers, especially with highly hydrophobic ones. Thus, extensive research studies have been conducted for the surface treatment of CNCs in order to overcome these problems [80]. 

CNFs, also known as nanofibrillar cellulose and cellulose nanofibers, possess hierarchical structures made up of interconnected fibrils with diameters ranging from 1 to 100 nm and an aspect ratio higher than 15 [81,82]. Top-down mechanical disintegration methods such as grinding, cryocrushing, high-intensity ultrasonication, and high-pressure homogenization are usually employed for the CNFs’ isolation. Through these techniques, dilute suspensions of cellulose fibers are subjected to high shear and impact forces, thus leading to mechanical cleavage along the longitudinal direction of the cellulosic source [78,81,82,83]. Specifically, the defibrillation methods yield nanostructures with both crystalline and amorphous regions. The high flexibility of CNFs is due precisely to the presence of the amorphous component. However, due to their large aspect ratio, the mechanical derived nanocelluloses are more susceptible to fiber agglomeration, which makes their further processing more challenging. 

Alternatively, BC, named bacterial nanocellulose, microbial cellulose, or bio-cellulose, is produced through a bottom-up approach using different aerobic non-pathogenic bacteria [82,83,84]. Besides being the purest form of nanocellulose, BC shows an ultrafine network structure containing fibers with micrometers in length and 20–100 nm in diameter, high water holding capacity and flexibility, and high crystallinity. Its outstanding physical, structural, and mechanical features make BC an ideal candidate for biomedical applications. 

**Figure 1:**
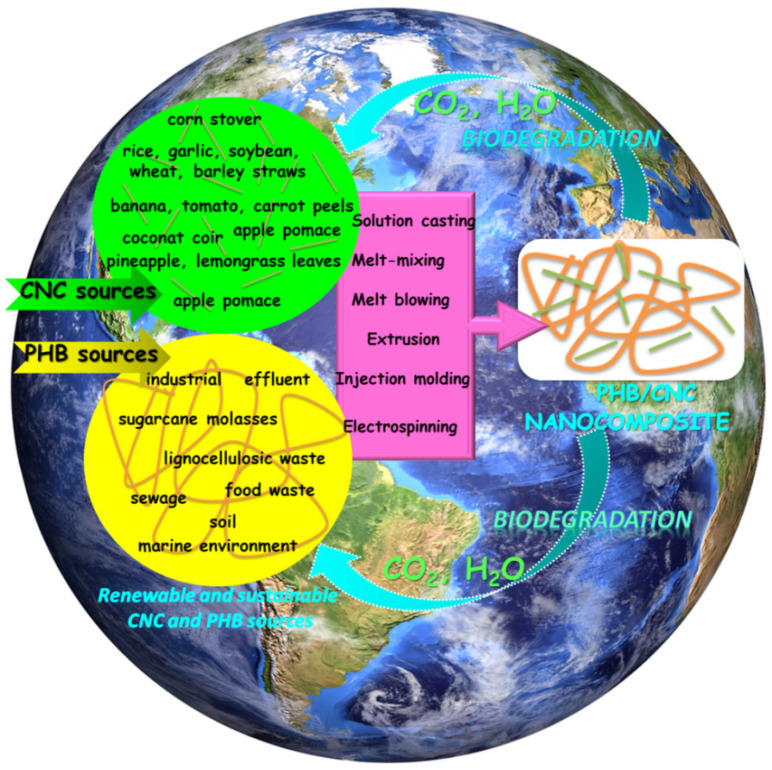
Circuit of biodegradable PHB/CNCs nanocomposites in the context of a circular economy.

Nanocelluloses, regardless of their source or preparation method, have been intensively studied as reinforcements in biopolymers for improving their mechanical and barrier properties [16,17,85,86]. The biocomposites from aliphatic polyesters and bacterial cellulose, as well as the nanocomposite materials from microfibrillated cellulose and hydrophilic or hydrophobic polymers, have been already reviewed [87,88]. However, much recent literatures on nanocellulose based materials are focused on biopolymers reinforced with CNCs. CNCs may be easily obtained both in labs and industrial facilities by employing less energy intensive processes as compared to CNFs. Due to the bio-origin of both PHB and CNCs and their subsequent biodegradation to carbon dioxide and water, the PHB/CNCs nanocomposites are of particular interest in the context of the world’s transition towards a circular economy (Figure 1). 

## 4. PHB Nanocomposites with Cellulose Nanocrystals

### 4.1. Preparation Routes

Solvent casting, melt processing, and electrospinning are the most used methods for obtaining PHB or PHBV nanocomposites with cellulose nanocrystals [89,90,91]. From these, solution casting is used more frequently due to some advantages such as its simplicity, easy application in lab conditions, and the obtaining of a good dispersion of the cellulose nanocrystals in the polymer matrix. However, this method has the drawback that it uses toxic solvents thatare difficult to be entirely removed from the samples. Moreover, the largely different character of cellulose and PHB, strongly hydrophilic vs. hydrophobic, and, therefore, the different solvents needed to dissolve the PHB and disperse the CNCs, make difficult the mixing of their solutions. Melt processing by using batch mixers, kneaders, or twin-screw extruders followed by injection or compression molding is advantageous because it can be easily transposed in industry, being an environmentally friendly method, which does not use dangerous solvents. However, the specialized equipment required for performing this process, the energy consumption needed for melting the PHB and for the mixing process, and the tendency of the cellulose nanocrystals to aggregate in the polymer melt are several disadvantages that must be overcome. In addition, PHB is sensitive to thermal degradation during melt processing [32,92]. Electrospinning has advantages related to easy scale-up, inexpensiveness and ability to incorporate various additives and polymers in the process, however it suffers from the same drawbacks as the solvent casting method, related to toxicity and difficult removal of the solvent. Additive manufacturing or reactive blending [93], processing under supercritical conditions or foaming [94] were also tried as new routes to obtain PHB nanocomposites with cellulose nanofillers. 

#### 4.1.1. Solution Casting

CNCs extracted from different sources (bamboo pulp, microcrystalline cellulose (MCC), or bleached pulp board) by sulfuric acid hydrolysis were used to obtain PHB/CNCs nanocomposites by solution casting using chloroform or dimethylformamide (DMF) as a solvent. Several methods were used to disperse the CNCs into the PHB solution: (i) a CNCs suspension in water was dispersed in acetone and then in chloroform through a sequential solvent exchange process consisting of a succession of dispersions and centrifugations, and further the CNCs dispersion in chloroform was mixed with a chloroform solution of PHB [89,95,96]; (ii) the CNCs were transferred from water to DMF using a solvent exchange method and then mixed with the PHB solution in DMF [77]; (iii) the water suspension of CNCs was lyophilized, and then the CNCs were redispersed in DMF and finally mixed with the DMF solution of PHB [86,97,98,99,100,101]. The concentration of CNCs in PHB was maintained at low values of up to 6 wt% in all the reported works. At a CNCs concentration below 2 wt%, a homogeneous dispersion of the CNCs in the PHB matrix was obtained, while at higher contents, CNCs agglomerations were observed with the naked eye as small white dots distributed in the transparent PHB matrix [95]. Due to their ability to scatter light, the CNCs agglomerates led to a decrease in the transparency of the PHB/CNCs nanocomposites. FESEM images showed that the CNCs were homogeneously dispersed into the PHB matrix at a CNCs content of 1 wt% [95]. Similar results were reported by Zhang et al. [89] which prepared PHB/CNCs and PHB/CNFs nanocomposites with a nanocellulose content of 1, 3, and 5 wt%, using a solution casting method. The SEM images showed that the best dispersion of CNCs in the PHB matrix was obtained at a CNCs loading of 1 wt%, while at CNCs contents of 3 and 5 wt%, a high tendency of CNCs to form agglomerates was noticed. This was due to the strong hydrogen bonds formed between the CNCs nanoparticles at these higher concentrations [89]. The transparency tests revealed that the transmittance of the PHB/CNCs and PHB/CNFs nanocomposite films decreased with the increase of CNCs and CNFs concentration. This was attributed to the poor compatibility between the hydrophilic nanocellulose and the hydrophobic PHB matrix and to the formation of nanocellulose agglomerates, which prevent light transmission [89]. When PHB/CNCs nanocomposites with 2, 4 and 6 wt% CNCs were compared to a PHB/2 wt% BC nanocomposite, all of them being obtained by solution casting, the transparency tests showed that the transmittance of the nanocomposites was higher as compared to that of pure PHB [86]. The best transparency was observed for the PHB/CNCs films due to the good CNCs dispersion and favorable interactions between the CNCs and the PHB matrix [98]. 

Several methods were applied to improve the properties of PHB/CNCs nanocomposites obtained by solution casting, such as the addition of plasticizers or the use of PHBV copolymer instead of PHB. Different plasticizers were added to the PHB/CNCs nanocomposities during the solution mixing and casting process to improve their flexibility and processability [99,101]. For example, PHB/CNCs nanocomposites with a CNCs loading of 2 and 4 wt%, respectively, and 20 wt% glyceryl tributyrate (TB) or poly [di (ethylene glycol) adipate] (A) as plasticizers were prepared via solvent casting using DMF as a solvent. TB addition led to PHB/CNCs nanocomposites with a lower thermal stability than that of pure PHB due to its easy evaporation and increased mobility of the polymer chains, which facilitated the diffusion of the decomposition products. On the contrary, the addition of plasticizer A led to PHB/CNCs nanocomposites with higher thermal stability than neat PHB due to the high molecular weight of A plasticizer which prevents its migration from the nanocomposites. CNCs addition was shown to have a beneficial effect on the stiffness and barrier properties of the plasticized nanocomposites, especially in the case of TB containing nanocomposites. This was explained by the good dispersion of the CNCs in the TB plasticized-PHB matrix due to the good compatibility between TB and the PHB matrix and the favorable hydrogen-bonding interactions established between TB and CNCs [99]. Therefore, the addition of TB plasticizer enhanced the PHB—CNCs interactions and improved the dispersion of the CNCs [101]. The decrease in the contact angle (CA) value for the PHB/CNCs nanocomposites as compared to pure PHB, regardless of whether CNCs or BC was used as reinforcing agent and whether A or TB was used as plasticizer, indicated an increased hydrophilicity. This may be assigned to various causes: (i) the plasticizing effect of TB or A, which led to an increased mobility of the PHB chains facilitating the diffusion of water into the material, (ii) the existence of numerous hydrophilic groups on the CNCs surface, which might have increased the hydrophilicity of the material or (iii) the poor dispersion of BC in the PHB matrix, which left a higher mobility to the PHB chains, favoring the diffusion of water molecules inside the material. However, CA values did not decrease below 65°, except for the PHB/4 wt% CNCs nanocomposite (A plasticizer) [101]. The PHB/CNCs nanocomposite films, containing 20 wt% TB, were applied as coatings on a cellulose paperboard by compression molding [100]. The CNCs from the nanocomposite films increased the interaction between layers as a result of the hydrogen bonding interactions between the hydroxyl groups from their surface and the OH groups of the paperboard, leading to enhanced mechanical properties. In addition, the PHB/CNCs layer improved the barrier properties of the paperboard, which became more suitable for packaging applications [100]. 

PHB/CNCs nanocomposites containing 15 wt% PEG and a low amount of CNCs (up to 0.75 wt%) were prepared by a solvent casting method [102]. PEG was used as a plasticizer and compatibilizer based on its miscibility with PHB and its affinity to cellulose due to the formation of hydrogen bonds between the carbonyl (-C=O) groups of PEG and the hydroxyl (-OH) groups of CNCs. The CNCs were first dispersed in PEG, the CNCs surface being covered by a PEG layer. Indeed, the TEM images revealed a homogeneous dispersion of the CNCs in the PHB matrix at a CNCs content of 0.15 wt%. This confirmed the ability of PEG to act as a coupling agent between PHB and CNCs. Based on the ATR-FTIR spectra and the electron microscopy images of the PHB/PEG/CNCs nanocomposites, it was supposed that (i) for a CNCs s content up to 0.45 wt% nearly the entire surface of the CNCs was covered by PEG, so that all or almost all the interactions between the PHB and the CNCs occurred preferentially via their PEG coating; (ii) for a higher CNCs content, when the PEG/CNCs ratio was low, the amount of PEG was no longer enough to cover the entire surface of CNCs, so that the interactions between PEG and PHB decreased, becoming more likely that the CNCs interacted directly with the PHB matrix. This model was supported by the variation of the mechanical properties of the nanocomposites, up to a concentration of 0.45 wt% the PEG-coated CNCs showing no reinforcing effect [102]. 

**Figure 2:**
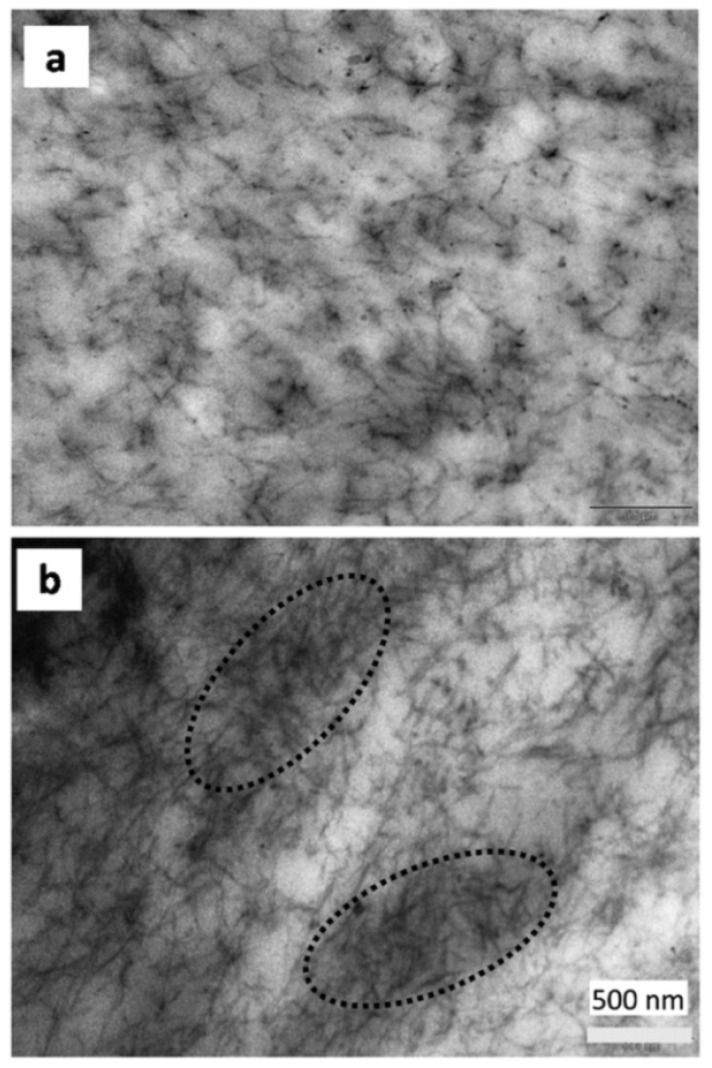
TEM images of the PHBV/CNCs nanocomposites with 2.3 wt% CNCs (**a**) and 4.6 wt% CNCs (**b**) Reprinted with permission from Ref. [97]. Copyright 2012 American Chemical Society.

The use of PHBV instead of PHB in the nanocomposites with CNCs was also tried as a method to improve the processability and flexibility of the nanocomposites [97,103]. Two methods were used to incorporate the CNCs in PHB. In one method [97], the gel-like CNCs, resulting from the sulfuric acid hydrolysis of microcrystalline cellulose (MCC), were freeze-dried. Then, the resulting powder was added to the PHBV solution in DMF, ultrasonicated and casted. Transparent films containing 1–5 wt% CNCs were thus formed [103]. In the second method [97], water suspensions with different concentrations of CNCs were added dropwise in DMF under stirring and, after the evaporation of water, PHBV was dissolved in the CNCs suspensions. PHBV/CNCs films with 0.5–4.6 wt% CNCs were obtained by solution casting, similar to the first method. The PHBV/CNCs films obtained by the two methods showed different thermal and mechanical properties [97,103]. Thus, a continuous decrease of the cold crystallization temperature (T_cc_) with the increase of the CNCs concentration was observed in the nanocomposites obtained by the first method and a decrease in T_cc_ only at loadings lower than 2.3 wt% in the second case. A similar trend was noticed for the variation of the mechanical properties, which indicated a more homogeneous dispersion of the CNCs in the nanocomposites obtained by the first method and a worse dispersion of CNCs at CNCs loadings exceeding 2.3 wt% when the solvent exchange-solution casting method was employed. The CNCs agglomerations were clearly observed in the TEM image of the PHBV/4.6 wt% CNCs film (Figure 2) [97]. 

#### 4.1.2. Melt Processing

Melt processing may be considered as the most important method to obtain PHB/CNCs nanocomposites due to the eco-friendliness and good fitting to the industrial processing techniques. However, the incorporation of CNCs in the PHB melt may be a difficult task due to the high tendency of hydrophilic CNCs to agglomerate in a hydrophobic environment. 

Chen et al. [104] used freeze-dried CNCs obtained by the sulfuric acid hydrolysis of MCC to prepare PHB/CNCs nanocomposites. A melt mixing method using a Haake Polylab Rheometer heated to 180 °C was employed to incorporate 2 wt% CNCs into the PHB and the resulted nanocomposite was subjected to crystallization studies to determine the CNCs effect on the PHB crystallization. Compared to MCC, CNCs had a higher influence on the spherulite morphology of PHB. CNCs acted as a heterogeneous nucleating agent causing an increase in the PHB crystallization rate simultaneously with a decrease in the energy barrier of PHB nucleation and in the folding surface free energy [104]. In addition, CNCs incorporation influenced the banded structure of the PHB spherulites, leading to a decrease in the average band space of the ring-banded spherulites. This was assigned to the increase in the crystallization rate of PHB in the presence of CNCs, which led to unbalanced stresses favoring the lamellae twist and the formation of ring-banded spherulites with reduced band space [104].

Jun et al. [90] used a PHBV matrix to prepare nanocomposites with two types of nanocelluloses (CNCs and CNFs) in different concentrations from 1 to 7 wt%. CNCs were obtained via the sulfuric acid hydrolysis of rice straws and CNFs resulted from the pressure-grinding of the cellulose extracted from the same source. For the preparation of nanocomposites, the PHBV powder was added to the nanocellulose suspensions under stirring and the mixtures were vacuum-dried for 24 h. A Haake co-rotating twin-screw extruder was used for melt compounding the mixtures. Both CNCs and CNFs showed a nucleation effect, accelerating PHBV crystallization and improving the Young’s modulus of nanocomposites, the optimum mechanical properties being obtained at 1 wt% CNCs [90]. 

#### 4.1.3. Other Methods

Electrospinning was used to obtain PHB/CNCs nanocomposite fibers with a content of CNCs of 5, 8, 12, 17, and 22 wt% [91]. CNCs were obtained by the sulfuric acid hydrolysis of alkali-treated and bleached corn husk [105]. A solvent exchange method was used to disperse the CNCs in achloroform/DMF mixture (90/10 volume ratio), which was employed as a solvent in the electrospinning process [91]. As revealed by SEM, the obtained PHB/CNCs nanocomposite fibers presented a uniform surface, without beads, regardless of the concentration of CNCs in the PHB matrix. A decrease of the PHB/CNCs nanocomposite fibers’ diameter was observed with the increase of CNCs concentration in nanocomposites. This was attributed to the increase in the conductivity of the electrospinning solution with increasing CNCs loading as a result of the negatively charged sulfate ester groups formed on the CNCs surface during sulfuric acid hydrolysis [91]. 

PHB/CNCs nanocomposite foams with 2, 3 and 5 wt% CNCs, obtained via the sulfuric acid hydrolysis of pulp fibers, were prepared using a nonsolvent-induced phase separation (NIPS) method [106]. Chloroform was used as solvent while tetrahydrofuran (THF) or 1,4-dioxane (Diox) was used as nonsolvents. In the NIPS process, the addition of a nonsolvent reduced the polymer−solvent affinity leading to a phase-separated polymer solution with one phase rich in polymer, representing the backbone of the gel, which was penetrated by the polymer-poor phase (in the nonsolvent) [106]. When THF was used as nonsolvent, CNCs accelerated both the PHB crystallization and the nanocomposites gelation, showing a nucleating effect. In contrast, when Diox was used as nonsolvent, CNCs incorporation led to a decrease in both PHB crystallization and nanocomposites gelation rate. This was due to the better dispersion of the CNCs in Diox than in THF, preventing the movement of the PHB chains and delaying the crystals’ growth. However, no significant differences between the degrees of crystallinity of the PHB/CNCs nanocomposites obtained using THF or Diox as nonsolvents were observed [106].

PHB/CNCs (4 wt%) nanocomposites obtained by solution casting using DMF as solvent were compression molded and further applied as a coating to cellulose paperboards, resulting in bilayer structures [107]. The PHB/paperboard ratio was varied between 5 and 20 wt%. The addition of CNCs improved the adhesion between the PHB layer and the cellulosic paperboard. The PHB/CNCs coatings decreased the water sensibility of the cellulose layer, leading to paperboard/PHB-CNCs bilayer composites suitable for packaging [107].

### 4.2. Methods Used to Improve the Compatibility in PHB/CNCs Nanocomposites

Different methods were applied to improve the compatibility between the strongly hydrophilic CNCs and the hydrophobic PHB matrix [108,109,110,111]. Dispersion agents and compatibilizers are one of the simplest and sometimes efficient additives for improving the compatibility in polymer nanocomposites with CNCs [112]. PEG is a hydrophilic polymer that is miscible with PHBV and, therefore, it was used as a compatibilizer in the PHBV/CNCs nanocomposites [108]. PHBV/CNCs nanocomposites with CNCs contents of 2 and 5 wt% were prepared using solution casting or extrusion blending [108]. In the first method, CNCs were coated with PEG by dispersing the mixture of CNCs and PEG powders in DMF, and then the PHBV solution in DMF along with the CNCs/PEG suspension in DMF were mixed and casted. In the second method, the freeze-dried CNCs/PEG powder was pre-mixed with PHBV and melt compounded using a co-rotating twin screw extruder followed by injection molding [108]. A very good dispersion of the CNCs in the PHBV matrix was obtained when the solvent casting method was used, also supported by the enhancement of the mechanical properties. However, despite the presence of the PEG compatibilizer, the CNCs could not be well dispersed in the PHBV during melt compounding, with effect on the mechanical properties, which decreased as compared to those of pure PHBV. A possible explanation for this behavior is that the high shear stress generated by the twin-screw removed the PEG coat from the CNCs surface and blended it with the PHBV matrix with which is compatible [108]. 

Another method proposed to improve the dispersion of the CNCs in PHBV and the compatibility between the two components is the chemical modification of CNCs by grafting PHBV onto their surface [110]. OH-terminated PHBV oligomers, prepared through transesterification using ethylene glycol in diglyme and dibutyltin dilaurate as a catalyst, were grafted on the surface of CNCs. The grafting reaction took place in anhydrous DMF using toluene diisocyanate (TDI) as a coupling agent. The ungrafted PHBV was removed by refluxing with chloroform [110]. PHBV-grafted CNCs (PHCNs) were used to obtain PHBV/PHCNs nanocomposites by solution casting, the content of modified CNCs ranging from 5 to 30 wt%. Most of the PHBV/PHCNs nanocomposite films showed a transparency similar to that of pure PHBV films. A decrease in the UV-vis transmittance with an increase in the PHCNs content was noticed; good results were obtained at PHCNs loadings up to 20 wt%, while a strong reduction in the transparency of the PHBV/PHCNs nanocomposites with 25 or 30 wt% PHCNs was observed. This was due to the formation of PHCNs agglomerates in the PHBV matrix at a higher content of modified CNCs [90]. The addition of PHCNs into PHBV led to a great increase in the mechanical properties for PHCNs contents of up to 20 wt%. This was due to the good adhesion between the components and the effective stress transfer at the PHBV-PHCNs interface [110]. 

CNCs grafted with polylactide (CNC-g-PLA) were prepared and used in PHB as a more compatible reinforcing agent [113]. CNCs resulted from the sulfuric acid hydrolysis of filter paper were grafted with polylactide by surface-initiated ring opening polymerization of L-lactide. The synthesis of CNC-g-PLA was carried out in 1-allyl-3-methylimidazolium chloride ionic liquid in the presence of catalytic amount of (dimethylamino)pyridine. Prior to the ring-opening polymerization of L-lactide, the CNCs were homogeneously acetylated. PHB nanocomposites with 2 wt% CNCs or CNC-g-PLA were obtained using a melt compounding method [113]. The calorimetric results showed a large influence of the CNCs treatment on the crystallization behavior of PHB. Untreated CNCs acted as a heterogeneous nucleating agent enhancing PHB crystallization. A different role was observed in the case of CNC-g-PLA which retarded the nucleation of PHB crystals and acted as an antinucleating agent during PHB crystallization [113]. Therefore, the PHB/CNCs nanocomposite exhibited a higher crystallization rate than neat PHB while the PHB/CNC-g-PLA nanocomposite presented a lower crystallization rate, showing that the crystallization behavior of PHB could be controlled by the CNCs’ treatment [113]. 

A one pot acid hydrolysis/Fischer esterification process was used to obtain CNCs surface modified with butyric acid, lactic acid, and their mixture in the presence of 37% HCl [109]. To ensure a good dispersion of modified CNCs in PHBV, the CNCs_butyrate, CNCs_lactate and CNCs_butyrate_lactate nanofillers were subjected to a solvent exchange sequence, from water, to ethanol, acetone, and finally, to chloroform. The suspensions of modified CNCs in chloroform were then mixed with the solution of PHBV in chloroform/tetrachloroethane (50/50) and casted [109]. Due to the similarity between the chemical structures of the lactate and butyrate ester moieties grafted on the CNCs surface and that of the PHBV matrix, the adhesion between the polymer matrix and the reinforcing agents was considerably improved and a homogeneous dispersion of the modified CNCs in the PHBV nanocomposites was observed. Consequently, the PHBV/CNCs_butyrate, PHBV/CNCs_lactate and PHBV/CNCs_butyrate_lactate nanocomposites with 2 wt% CNCs showed a considerably improved transparency as compared to the nanocomposites containing unmodified CNCs [109]. However, the dynamical mechanical analysis results confirmed an improved interface only in the PHBV/CNCs_butyrate nanocomposites due to the similarity between the butyrate moieties attached on the CNCs surface and the PHBV matrix, which led to a better dispersion of the CNCs in the polymer matrix.

**Figure 3:**
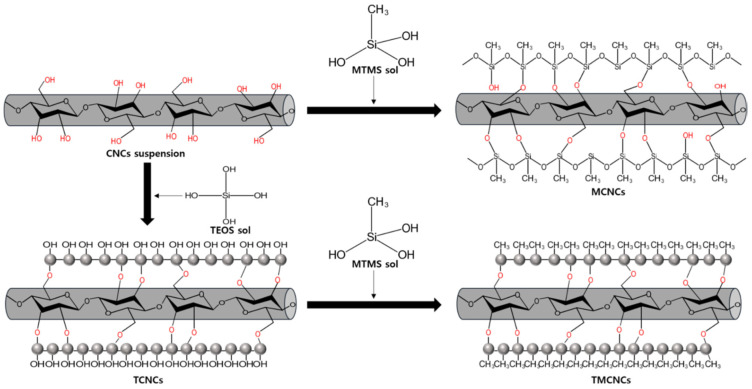
Schematic representation of the surface treatment of CNCs using a double silanization process [111].

A mixture of PHB and poly(4-hydroxybutyrate), denoted as PHB/P4HB, was reinforced with surface hydrophobized CNCs, which were obtained by a double silanization process, as shown in Figure 3 [111]. 

To improve the compatibility between the CNCs and the hydrophobic matrix, the CNCs were modified with (i) methyltrimethoxysilane (MTMS) resulting CNCs with a hydrophobic surface (MCNCs), (ii) tetraethyl orthosilicate (TEOS) resulting CNCs with spherical SiO_2_ nanoparticles on their surface (TCNCs) and (iii) TEOS then MTMS, resulting TCNCs with CH_3_ ends (TMCNCs). The three types of modified CNCs were melt-compounded with PHB/P4HB by extrusion followed by compression molding resulting nanocomposite plates. MCNCs and TCNCs showed a low compatibility with PHB/P4HB and many aggregated nanocrystals were observed in the nanocomposites with 10 wt% modified celluloses. On the contrary, freeze-dried TMCNCs showed a homogenous dispersion in the PHB/P4HB matrix and no nanocrystals agglomeration [111]. 

### 4.3. Nanocomposites from PHB Blends and CNCs

The addition of a third polymer in PHB/CNCs nanocomposites was also used as a method to improve the compatibility and the properties of PHB/CNCs nanocomposites [114,115,116,117]. Based on the compatibility of poly(vinylacetate) (PVAc) and PEG with PHB or PHBV and their hydrophilic character, similar to that of CNCs, the two polymers were used as a third component in PHB/CNCs nanocomposites [114]. PHB/PVAc/CNCs, PHB/PEG/CNCs, PHBV/PVAc/CNCs, and PHBV/PEG/CNCs ternary nanocomposites with 2.4 or 4.8 wt% CNCs were prepared by melt mixing PHB or PHBV with PVAc/CNCs and PEG/CNCs masterbatches in a Haake double-screw mini-extruder. The masterbatches were prepared by dispersing the CNCs into a PVAc water emulsion or into a PEG solution in water, followed by solution casting as films and drying [114]. Due to the partial miscibility of PVAc or PEG with PHB and PHBV, they improved the dispersion of the CNCs into the polymer matrix. This was determined by the favorable hydrogen bonding interactions between the hydroxyl groups on the CNCs surface and the polar groups on the PVAc and PEG chains. The addition of a third polymer had as a result an improvement in the mechanical properties, more important in the case of the PVAc-containing nanocomposites. The plasticizing effect of PEG could be a cause of the lower improvement in the mechanical properties observed in the PEG-containing nanocomposites [114]. 

A combined solvent casting and melt processing technique was used to obtain a good dispersion of CNCs from pine cones in a PHB/poly(ε-caprolactorne) (PCL) blend [115]. CNCs were dispersed in chloroform and were added in a mixture of PHB/PCL (75:25) in chloroform under intense stirring. The solvent casted films were melt-compounded in a twin screw microextruder and compression molded. Nanocomposites with a content of CNCs of 3, 5 and 7 wt% were obtained by this method. In low amounts, CNCs enhanced the compatibility between the immiscible PHB and PCL and reduced their phase separation during the melt blending. This was due to the tendency of CNCs to locate at the PHB - PCL interface preventing the coalescence of the dispersed PCL phase in the continuous PHB matrix during the melt processing. The best dispersion of CNCs in the PHB/PCL matrix was obtained for a CNCs content of 3 wt%. In this case, the nanocomposite showed a significant increase in transparency that can be attributed to the good dispersion of CNCs and their compatibilization effect, which increased the PHB-PCL miscibility [115].

Poly(lactic acid) (PLA) and CNCs were added in a PHB plasticized with epoxidized canola oil (eCO) to improve its mechanical properties [116]. The PHB/PLA/eCO/CNCs nanocomposites with a PHB:PLA weight ratio of 3:1 and 5 wt% CNCs (related to the PHB/PLA amount) were obtained by melt-mixing using a conical twin-screw micro-extruder. The eCO green plasticizer was added to increase the flexibility and thermal stability of PHB, both important drawbacks of this biopolymer. The concomitant addition of PLA and eCO proved to be beneficial to both the elastic properties and thermal stability of nanocomposites [116]. 

A melt compounding masterbatch technique was applied to obtain PLA/PHB/CNCs nanocomposite films plasticized with a low content of acetyl tributyl citrate [117]. In addition to being an easily scalable and environmentally friendly technique, the melt compounding masterbatch technique ensured a good dispersion of the CNCs and plasticizer in the PLA/PHB blend. This technique consists in the fabrication of a PHB masterbatch, containing both the plasticizer and the CNCs, and its further dilution in PLA, both phases comprising melt compounding operations. The addition of 5 wt% CNCs to the plasticized PLA/PHB matrix led to an increase in the storage modulus due to the stiffening effect of the CNCs and the good dispersion of the high aspect ratio CNCs in the polymer matrix [117]. 

## 5. Influence of CNCs on the Properties of PHB/CNCs Nanocomposites

### 5.1. Thermal Properties

The crystallization behavior of PHB is very important because it determines many of its properties. PHB is characterized by a high crystallinity and a low crystallization rate which has as result the appearance of large spherulites. The addition of CNCs to PHB has a strong influence on its crystallization behavior and its crystalline structure. Thus, CNCs acted as nucleating agents, facilitating the PHB crystals growth and leading to the formation of greater lamellar thickness spherulites during the casting process [98]. This was proved by an increase in the melting temperature (T_m_) of PHB following CNCs addition, observed in the differential scanning calorimetry (DSC) scans. A slight increase in the melting temperature of PHB, similar to that determined by the CNCs, was observed in nanocomposites with different nanocelluloses [86,99]. Thus, an increase of T_m_ with about 4 °C was recorded for PHB/CNFs nanocomposites with a CNFs loading of 5 wt% [89]. 

The nucleating effect of CNCs was also observed through the increase in the crystallization temperature (T_c_) of PHB from around 60 °C for the pure PHB to approximately 83 °C for the PHB/CNCs nanocomposites [98]. The concentration of CNCs in PHB nanocomposites also influenced the T_c_. An increase of T_c_ with about 18 °C was noticed for the PHB/CNCs nanocomposite with 1 wt% CNCs compared to neat PHB, showing the nucleating effect of CNCs that reduced the nucleation energy, increasing the PHB nucleating rate and facilitating its crystallization [89]. However, a lower increase of T_c_ with only 7.6 °C was observed at a CNCs loading of 5 wt% in nanocomposite. This was due to the CNCs tendency to form aggregates in the PHB matrix when incorporated in higher concentrations, which decreased their nucleating effect [89].

A low influence of CNCs on the degree of crystallinity of PHB was in general reported [86,98]. A slight increase in crystallinity (X_c_) was noticed in PHB/CNCs nanocomposites with 5 wt% CNCs as compared to the pure PHB and the nanocomposites with lower content of CNCs [89]. The lower crystallinity of PHB/1 wt% CNCs nanocomposite was determined by the homogeneous dispersion of the rigid CNCs in the PHB matrix, which obstructed the segmental movement of the PHB chains, hindering the PHB crystallization [89].

The addition of a plasticizer in the PHB/CNCs nanocomposites may lead to an increase in the degree of crystallinity [100,101]. Thus, an increase in the X_c_ from 55.4% for the pure PHB to 62.7% for the PHB/CNCs nanocomposite containing 4 wt% CNCs and 20 wt% low molecular weight tributyrin as plasticizer was observed [101]. Therefore, the nucleating activity of CNCs was enhanced when TB was used as plasticizer. Similarly, the addition of 10 wt% epoxidized canola oil plasticizer in the PHB/PLA blend favored the increase of crystallinity, from 23.5% in the blend to 42.4% in the plasticized blend, although the addition of CNCs to the plasticized PHB/PLA blend did not lead to a further increase in the crystallinity degree [116]. This was supposed to be due to the poor dispersion of the CNCs in the PHB/PLA blend, which prevented the nucleation and crystallization. 

The surface modification of CNCs also led to a reduction in the degree of crystallinity [110,113]. This was observed in the PHB nanocomposite with CNC-g-PLA vs. the nanocomposite containing untreated CNCs [113], as well as in the case of PHBV/PHCNs nanocomposites, where the CNCs grafted with PHBV were well dispersed in the PHBV matrix [110]. The treatment of CNCs ensured a good entanglement between the chains of the PHBV matrix and those grafted on the cellulose nanocrystals, as well as the formation of numerous hydrogen bonds interactions between the PHBV matrix and PHCNs. All these hindered the PHBV crystallization, leading to the formation of imperfect PHBV crystals and to a reduction in the crystallinity degree [110]. This idea was supported by the progressive increase in the glass transition temperature of the nanocomposites with the increase of PHCNs content that was assigned to the numerous hydrogen bonds and entanglements formation, which prevented the segmental movement of the PHBV chains, making the PHB/PHCNs nanocomposites more rigid as compared to the pristine PHBV [110]. 

The thermal stability of cellulose nanofillers strongly depends on the methods used to obtain them. Acid hydrolysis of cellulose using concentrated acids and, especially, sulfuric acid, is a harsh treatment leading to the degradation of the disordered amorphous regions and a strong decrease of the degree of polymerization of cellulose [118]. As a result, the good thermal stability of pure cellulose, which begins to decompose after 300 °C [119], may be strongly decreased, with an effect on the thermal stability of PHB. Different influences of CNCs on the thermal stability of PHB were reported [89,90,98]. Thus, a decrease in the thermal stability of PHB, corresponding to a reduction in the temperature of the maximum degradation rate (T_d_) of around 20 °C, was observed after CNCs’ addition [98]. This was considered as an effect of the sulfate groups on the CNCs surface, which induced and accelerated the scission of the PHB polymer chains. On the contrary, an improved thermal stability consisting of an increase in T_d_ from 286.5 °C for pure PHB to 291.7 °C for the nanocomposite with 5 wt% CNCs was observed [89]. A similar increase was reported for the PHBV/CNCs nanocomposites, from 278.6 °C for the pure PHBV to 284.3 °C for the nanocomposites with 7 wt% CNCs [90]. This was assigned to the hydrogen bonds formed between the carbonyl groups of PHB and the hydroxyl groups of CNCs, which slowed down the random chains breaking in PHB, improving its thermal stability [89]. Although no information on the thermal stability of the CNCs was disclosed in these two studies [89,98], the different processes used to obtain the CNCs and to incorporate them in the PHB matrix could influence these results. Thus, it is possible that the solvent exchange process [89] could contribute to a better thermal stability of the CNCs than the freeze drying process [98] along with the different solvents used in the solvent casting processes, requesting different conditions for their removal. 

A different thermal stability of the PHB/CNCs nanocomposites depending on the concentration of CNCs was also reported [95]. Thus, a better thermal stability was observed for the nanocomposite with 3 wt% CNCs as compared to the pure PHB but a poor one for the nanocomposite with 5 wt% CNCs. Kinetic studies on the thermal degradation process of the nanocomposites showed that the rate of degradation decreased with an increase in the CNCs content up to about 3 wt% and increased with the further increase of CNCs concentration in nanocomposites [95]. This complex behavior was explained by the fact that, at CNCs concentration of 3 wt%, a good dispersion of the CNCs in the PHB was obtained. In this case, the formation of hydrogen bonds between the nanofiller and the polymer matrix hindered the movement of the chain segments of PHB, which impeded the diffusion of volatile products through the nanocomposites, enhancing their thermal stability. Conversely, the formation of CNCs aggregates in the nanocomposites with 5 wt% CNCs altered the thermal stability of PHB as they are believed to act as nucleation sites for the chain scission degradation of PHB at higher temperatures [95]. It was concluded that the thermal degradation mechanism of the PHB/CNCs nanocomposites followed first order reaction and phase boundary controlled reaction models with a random chain scission mechanism characteristic of most polyesters. 

Although the addition of plasticizers generally decreased the thermal stability of PHB due to their high volatility, which reduced the onset degradation temperature [101], a favorable influence was observed after the addition of 15 wt% PEG to PHB/CNCs nanocomposites with low content of nanocrystals [102]. The highest increase in the T_d_, of about 27 °C, was obtained for the PHB/PEG/CNCs nanocomposite with 0.45 wt% CNCs. This was assigned to the better adhesion between the CNCs and PHB matrix, ensured by the PEG coating which, at this CNCs concentration, covers almost the entire surface of the CNCs [102]. It has been shown that the presence in the nanocomposites of a high molecular weight poly(adipate diethylene) plasticizer instead of a low molecular weight one (TB), in a 20 wt% concentration relative to PHB, considerably increased the maximum degradation temperature compared to the pure PHB, PHB/CNCs nanocomposites and the nanocomposites containing the same amount of TB plasticizer [101].

Surface functionalization of CNCs had also a favorable influence on the thermal stability of PHBV [110]. In particular, CNCs grafted with PHBV (PHCNs) strongly increased the thermal stability of PHBV, an improvement in the T_d_ of about 35 °C being noticed at a concentration of PHCNs in PHBV of 20 wt%. This improvement was explained by the strong interactions established between the functionalized CNCs and the PHBV matrix as well as the entanglements formed between the PHBV chains of the polymer matrix and the PHBV chains attached to the CNCs surface. Due to the significant increase in the initial decomposition temperature, which approached 250 °C, all PHBV/PHCNs nanocomposites showed higher melt-processing windows as compared to the neat PHBV matrix [110]. However, only a slight increase of T_d_ was observed after the addition to PHBV of CNCs surface modified with butyric acid, lactic acid, and their mixture [109]. On the contrary, the incorporation of CNCs into the PHB/PCL blends led to a significant decrease in the thermal stability of PHB, probably due to the lower thermal stability of CNCs [115]. The most important thermal properties of PHB/CNCs nanocomposites are summarized in Table 1.

### 5.2. Mechanical Properties

The reinforcing effect of CNCs in PHB nanocomposites is based on the remarkable mechanical properties of cellulose nanofillers [120,121]. The Young’s modulus of a cellulose whisker calculated from the shift in the characteristic band at 1095 cm^−1^ in the Raman spectrum was 143 GPa [120], while the transverse Young’s modulus of a nanocellulose film determined by PeakForce Atomic Force Microscopy was 19 GPa [121]. Similar to other biopolymers, the mechanical properties of PHB are weaker than those of most common thermoplastic polymers; therefore, the addition of cellulose reinforcements can bring significant gains in stiffness and other mechanical properties. However, the mismatch between the PHB hydrophobicity and the strong hydrophilicity of CNCs prevents the good dispersion of CNCs in the polymer matrix and reduces the efficiency of the load transfer at the polymer–fiber interface. As a result, the addition of CNCs in PHB did not always lead to a strong improvement in strength and stiffness [90,106,107]. 

The addition of CNCs in PHB generally determined a continuous increase in the Young’s modulus with an increase in the CNCs concentration in nanocomposites [89,98]. This effect was assigned on the one hand to the rigid nature of the CNCs, which potentiated the PHB stiffness and increased the degree of crystallinity of PHB when present in higher amounts in the PHB matrix [89] and, on the other hand, to the homogeneous dispersion of the CNCs in the PHB matrix due to the possible hydrogen bonding interactions between CNCs and PHB [86]. A different effect of CNCs on the tensile strength of PHB/CNCs nanocomposites was reported, depending on the dispersion of the CNCs in the PHB matrix. A continuous improvement in the tensile strength of PHB with increasing CNCs content between 2 and 6 wt% was observed in nanocomposites with a homogeneous dispersion of the CNCs [98] while a decrease in the tensile strength with increasing CNCs content from 1 to 5 wt% was achieved for the nanocomposites where CNCs agglomerates were pointed out by SEM [89]. This may be due to the fact that CNCs agglomerates acted as stress concentrators from where the cracks were initiated. The presence of rigid CNCs in higher concentration in the PHB matrix and possible fibers agglomeration were also reflected in a decrease of elongation at break [89,98]. Curiously, an increase of the elongation at break was observed in the nanocomposite with 1 wt% CNCs [89]. At low CNCs loadings, the homogeneous dispersion of the CNCs in the PHB matrix led to a restriction of the segmental movement of the PHB chains and impeded the PHB crystallization causing an increase in the elongation at break [89]. 

A different mechanical behavior was reported for the PHBV/CNCs nanocomposites [90,97]. In nanocomposites containing 0.5–4.6 wt% CNCs, an increase in the tensile strength and Young’s modulus with an increase in the CNCs concentration, followed by a leveling off from a content of CNCs of 2.3 wt%, was noticed [97]. Therefore, the good dispersion of CNCs in the PHBV matrix at loadings of up to 2.3 wt% ensured a large interface area between PHBV and CNCs and the enhancement of the mechanical properties, while the CNCs agglomerates, which were formed at higher concentrations of CNCs, were probably the main cause of the mechanical properties’ decline after the 2.3 wt% concentration threshold [97]. In PHBV/CNCs nanocomposites with 1–7 wt% CNCs obtained by melt compounding [90], the increase in the concentration of CNCs determined a gradual increase in the Young’s modulus, along with a gradual decrease in the tensile strength and elongation at break. The decrease in the tensile strength highlighted the lack of adhesion between CNCs and PHBV, while the drop in elongation showed the reduced deformability at PHBV/CNCs interface [90].

The influence of CNCs in large amounts, from 5 to 22 wt%, on the mechanical properties of PHB/CNCs nanocomposite fibers prepared via electrospinning highlighted the presence of a more favorable concentration of CNCs for which the improvement of the mechanical properties of the nanocomposite fibers was maximum [91]. Accordingly, the largest improvement in tensile strength (by 13%) and elastic modulus (by 6%) was observed for a content of CNCs of 8 wt%. The formation of CNCs agglomerates which acted as stress concentrators and the weaker PHB-CNCs interface were considered responsible for the decrease in the mechanical properties of the nanocomposite fibers with high CNCs content [91].

The addition of a plasticizer together with CNCs was also tested as a method to improve both the flexibility-processability and the stiffness of PHB [99,102]. However, in most cases, no important changes or a decrease in the mechanical properties was obtained due to the diametrically opposite effects of the plasticizer and the reinforcing agent: while the reinforcing agent improves the PHB’s stiffness, increasing its elastic modulus and tensile strength and reducing its elongation at break, the plasticizer increases the elongation at break at the expense of the stiffness. A small variation of the Young’s modulus, along with a slight reduction in the tensile strength and strain at break, were observed for the PHB/TB/CNCs nanocomposites containing 20 wt% glyceryl tributyrate and 2 or 4 wt% CNCs as compared to neat PHB [99]. The highest values of Young’s modulus and tensile strength were obtained for the PHB/TB/CNCs composite with 4 wt% CNCs, showing that TB may enhance CNCs dispersion in the PHB matrix. This was due to the good compatibility between TB and PHB and the hydrogen bonding interactions between TB and CNCs. However, using a high molecular weight plasticizer such as poly [di (ethylene glycol) adipate] instead of TB in these nanocomposites had a detrimental effect on the mechanical properties, probably due to the lower miscibility between this plasticizer and the PHB matrix which didn’t allow a good dispersion of CNCs in PHB [99]. When the PHB/TB/CNCs nanocomposite film was used to obtain PHB/TB/CNCs-paperboard bilayer composites with a PHB content of 15 wt% related to the paperboard weight, 20 wt% TB plasticizer and 4 wt% CNCs relative to the PHB weight, an improvement in the tensile strength, Young’s modulus, and elongation at break compared to neat PHB was obtained [100]. In addition, peeling tests were undertaken to assess the adhesive strength between layers, the paperboard and the PHB/TB/CNCs film. During these tests, the PHB/TB/CNCs layer was peeled off from the paperboard surface with broken cardboard fibers adhering to its surface. This indicated that the PHB/TB/CNCs-paperboard adhesion was higher than resistance of the fibers in the paperboard due to the PHB penetration between the paperboard fibers during the compression molding [100]. 

**Figure 4:**
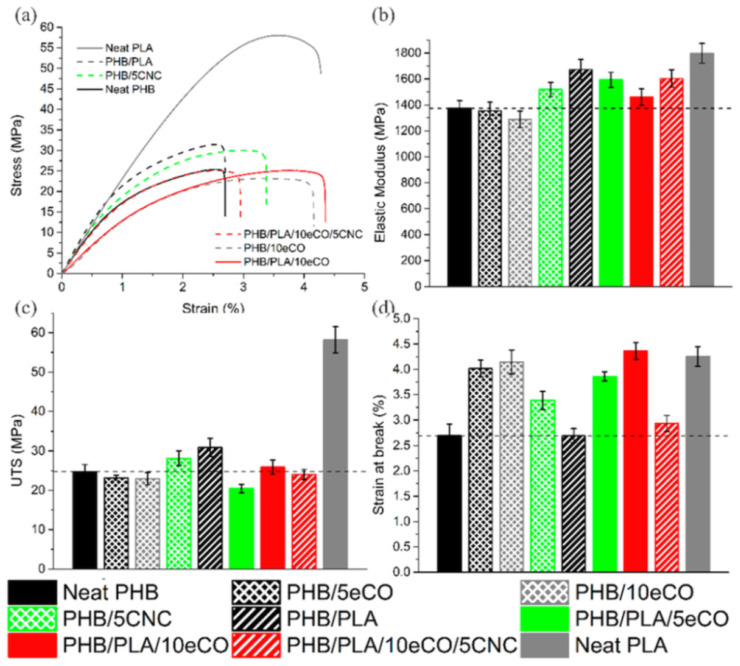
(**a**) Stress–strain curves of PHB blends and nanocomposites (**b**) Elastic modulus (**c**) Tensile strength at break, and (**d**) Elongation at break for neat PHB, neat PLA, PHB/5eCO and PHB/10eCO, PHB/PLA, PHB/PLA/5eCO and PHB/PLA/10eCO blends and nanocomposites (PHB/CNCs and PHB/PLA/10eCO/CNCs) [116].

A special effect was reported in the case of PHB/PEG/CNCs nanocomposites containing PEG plasticizer (15 wt%) and low amounts of CNCs (0.022–0.75 wt%) [102]. The addition of CNCs in PHB/PEG led to a significant reduction in both tensile strength (from 30 MPa to 22 MPa) and elastic modulus (from 1180 MPa to 537 MPa) of PHB, simultaneously with an increase in the elongation at break. The most important increase of flexibility was noticed in the nanocomposites with 0.45 wt% CNCs, where the total surface of CNCs was entirely covered by PEG and a 25-fold increase in the elongation at break was obtained [102]. Therefore, the CNCs slippage and the alignment of the PHB macromolecules during the plastic deformation can activate the shear flow of the polymer matrix, so that the materials can withstand greater deformations without a rapid catastrophic fracture. As a result, CNCs may act as a reinforcing agent only for higher amounts of CNCs in PHB/PEG [102]. Similarly, a decrease in the tensile strength of the PHB/PLA/eCO/CNCs nanocomposites of about 23% and a slight increase of the Young’s modulus of about 10% as compared to the PHB/PLA blend were observed after the addition of epoxidized canola oil (10 wt%) as a plasticizer and CNCs (5 wt%) as a reinforcing agent in a PHB/PLA (3:1) blend (Figure 4) [116]. The small efficiency of the CNCs on the mechanical properties of the plasticized blend was attributed to the inhomogeneous dispersion of CNCs in the presence of the eCO plasticizer and the formation of CNCs agglomerates which initiated the rupture of the material [116].

When PHBV-grafted CNCs were used as reinforcing agents in PHBV-based nanocomposites, a strong enhancement of the mechanical properties of PHBV, consisting in an increase in the tensile strength, Young’s modulus, and elongation at break, was reported over the entire concentration range [110]. This increase was attributed to the surface modification of CNCs, which ensured a good adhesion between PHCNs and PHBV both due to the entanglements between the chains of the PHBV matrix and the PHBV chains grafted on the CNCs surface, and the formation of hydrogen bonding interactions between the PHBV matrix and PHCNs. The largest improvements in the tensile strength, Young’s modulus, and elongation at break, by 113%, 95%, and 17%, were obtained for the PHBV/PHCNs nanocomposite with 20 wt% PHCNs [110]. A less pronounced reinforcing effect on the mechanical properties of PHBV was obtained when the CNCs were surface modified with butyric acid, lactic acid, or their mixture [109]. In this case, an improved storage modulus at temperatures higher than the T_g_ of PHBV was reported only for the nanocomposites containing butyrate-modified CNCs, where the improved interface between PHBV and CNCs_butyrate led to increased T_g_ and tan δ values [109]. The shift of the glass transition and the improved dumping showed a better stress transfer between the PHBV matrix and CNCs_butyrate determined by the good dispersion of CNCs_butyrate in the PHBV matrix, and to the similarity between the butyrate moieties attached on the CNCs surface and the PHBV matrix [109]. 

A different effect of CNCs on the mechanical properties of a PHB/P4HB mixture was reported after the surface treatment of CNCs with silanes [111]. The addition of 5 and 10 wt% freeze-dried double-silanized CNCs (TMCNCs) in PHB/P4HB determined a strong increase of the elongation at break along with a gradual decrease of the tensile strength and a decrease of the Young’s modulus only for the higher content of TMCNCs in nanocomposites. The strong increase of the flexibility was due to the action of TMCNCs as a plasticizer and nucleating agent, promoting the formation of smaller spherulites and enhancing the dispersibility of the surface treated CNCs in the polymer matrix [111]. The most important mechanical properties of PHB/CNCs nanocomposites are summarized in Table 1.

### 5.3. Barrier Properties

The barrier properties against water, light, gases or volatiles are essential when the PHB/CNCs nanocomposites are intended for food packaging application. Compared to other biopolymers, PHB shows better barrier properties due to its hydrophobicity, high crystallinity, and stereo-regularity [10], and the addition of CNCs may further improve these properties. Migration studies using nonpolar and polar food simulants were carried out on PHB/CNCs (1–5 wt%) nanocomposite films obtained by solvent casting [96] in order to determine if these substances can pass by diffusion or adsorption to a potential food product that was packaged using these nanocomposite films. The PHB/CNCs nanocomposites were subjected to migration studies according to the Commission Regulation EU No 10/2011: (i) in ethanol 10% (*v*/*v*) at 40 °C for 10 days and (ii) in isooctane at 20 °C for 2 days. When ethanol was used as a food simulant, the overall migration of the PHB/CNCs nanocomposites decreased as compared to the pristine PHB at low CNCs loadings, of 1 and 2 wt%. However, at CNCs loadings greater than 3 wt%, the migration increased significantly, reaching a maximum value for the nanocomposites having 5 wt% CNCs. At low CNCs contents, the strong interactions between the CNCs and PHB, which allowed a good dispersion of the CNCs in the PHB matrix and led to restrictions of the movement of the polymer chains, hindered the migration in ethanol. At higher CNCs contents, CNCs tended to agglomerate, and the adhesion between CNCs and PHB was considerably decreased, which left free way for the migration of the nanocomposites’ components in the simulant. The same allure of the migration levels as in the case of ethanol was observed when isooctane was used as food simulant, although the overall migration was much reduced [96]. The maximum migration values of 175 μg/kg in ethanol and 40 μg/kg in isooctane, obtained for the nanocomposites with 5 wt% CNCs, remained, however, far below the standard migration limits of 60 mg/kg established by the EU current legislation. Therefore, the migration tests showed that the PHB/CNCs nanocomposites are safe for using as food packaging materials [96]. 

**Figure 5:**
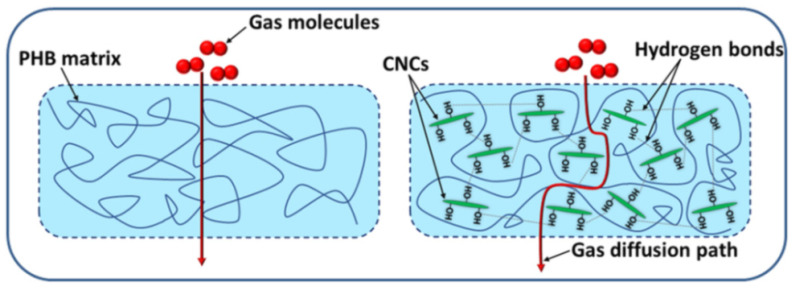
A schematic representation of the tortuous gas diffusion path in the case of a PHB/CNCs nanocomposite compared to pristine PHB.

When the PHB/CNCs nanocomposites are intended for packaging fresh produce, a high barrier to oxygen is important [10]. It was observed that the incorporation of low CNCs loadings, of 1 and 2 wt%, into the PHB matrix led to a significant reduction in the oxygen transmission rate (OTR), with 46% and 65% [96]. This was ascribed to the good PHB/CNCs interface established as a result of the hydrogen bonding between CNCs and PHB and the formation of a CNCs network inside the polymer matrix, which reduced the permeation of oxygen through the PHB/CNCs nanocomposites [96]. Moreover, a three-times reduction of the OTR was obtained in PHB/CNCs nanocomposites containing 3 wt% CNCs [89]. A schematic representation of the tortuous gas diffusion path in the case of a PHB/CNCs nanocomposite compared to pristine PHB is shown in Figure 5.

Good water vapor barrier properties are required for the PHB/CNCs nanocomposites intended to be used in the food packaging industry in order to prevent premature loss of flavor or spoilage of the food products [89]. PHB is characterized by a higher water vapor permeability (WVP) as compared to that of common polymers used in packaging, which may raise problems when baked products are packaged [10]. However, the addition of CNCs in PHB reduced its WVP [86,89,98]. Accordingly, a three-times reduction in WVP was reported for PHB/3 wt% CNCs nanocomposite as compared to neat PHB, although no further decrease was observed for a higher content of CNCs in the nanocomposites [89]. In the case of the PHB/CNCs nanocomposites obtained by solvent casting, a similar decrease in the WVP was reported after the addition of 2 wt% CNCs in PHB and no variation in the WVP value for higher CNCs amounts up to 6 wt% [98]. The decrease of the water vapor permeation through the nanocomposites films was determined by the CNCs, which acted as physical barriers hindering the diffusion of water molecules [89]. The differences observed in the variations of the WVP with the amount of CNCs may be determined by the good dispersion vs. agglomeration of CNCs, the adhesion to the PHB matrix, porosity of the films, and other factors [98]. Moreover, it was observed that the addition of a plasticizer (20 wt% TB) increased the water vapors permeation through the PHB/CNCs films, thus reducing their ability to protect the packaged food from humidity during shelf life [99]. In such nanocomposites, the plasticizer increased the free volume in the polymer, favoring water vapors diffusion and reducing the moisture barrier properties of the nanocomposites, while the CNCs acted as a barrier, increasing the tortuosity of the water vapor diffusion path in the materials [99] (Figure 5). A significant reduction in the water vapor permeability of the cellulose paperboard, with around two orders of magnitude, was observed after coating it with plasticized PHB/CNCs [100]. This was assigned to the CNCs homogeneously dispersed in the PHB matrix, acting as physical barriers to the diffusion of water vapor molecules.

An important characteristic of the PHB/CNCs nanocomposites intended for the food-packaging industry is their transparency, which allows seeing the packaging content. The transmittance of the films determined by UV-visible spectroscopy is an important characteristic showing not only the dispersion of CNCs in the PHB matrix but also the compatibility of the phases in the composite material [89]. In general, well dispersed CNCs should not significantly influence the transparency of the PHB. Indeed, in low concentration, CNCs did not change the transmittance of the PHB film; however, from a content of 5 wt% CNCs [89] or 2 wt% CNCs [96] in the nanocomposites their transparency declined. The surface treated CNCs allowed the addition of higher contents of CNCs in PHBV without a significant decrease in the UV-visible transmittance, [110] or even an increase in transparency compared to neat PHBV [109]. An increase of transparency was also reported after the addition of CNCs in the PHB/PCL blend [115].

### 5.4. Biodegradability

PHB is biodegradable in soil and water according to following ISO and ASTM biodegradation standards: ISO 14855 (controlled industrial and home composting conditions), ISO 15985 (anaerobic digestion), ISO 17556 (soil biodegradation test), ISO 14851 (biodegradation in aerobic, aquatic conditions), ISO 14853 (aqueous anaerobic biodegradation test) and ASTM D6691 (marine biodegradation) [122]. 

It was observed that coating cellulose paperboard with PHB/CNCs nanocomposites did not significantly change the biodegradation rate of paperboard under composting conditions [100]. Thus, the plasticized PHB/CNCs-cellulose paperboard bilayer composites showed a biodegradation behavior quite similar to that of the neat paperboard, a disintegration degree higher than 90% being achieved six weeks after they were incubated for biodegradation under composting condition. However, in the fifth week of the biodegradation test, a certain decrease in the biodegradation rate was observed for the plasticized PHB/CNCs-paperboard composites as compared to the uncoated paperboard. This was determined by the increased crystallinity degree of PHB and the formation of higher ordered PHB crystals following the CNCs and TB addition, which were much more resistant to biodegradation than the amorphous regions in the material [100]. Similarly, the addition of CNCs in PHB did not affect the good biodegradability of PHB [101]. Thus, the PHB/CNCs nanocomposites were disintegrated in a high proportion (90%) in about 21 days under composting conditions. At a low CNCs loading of 2 wt%, the PHB/CNCs nanocomposite containing TB plasticizer showed a lower degradation rate compared to pristine PHB in the first 14 days. This was probably due to the stiffening effect of the CNCs, which hindered the diffusion of water into the material, slowing down the attack of microorganisms, together with the higher PHB crystallinity, determined by the addition of the CNCs. As the CNCs loading increased to 4 wt%, the nanocomposites presented a much higher degradation rate, even in the first 14 days. This was due to the increased hydrophilicity and water absorption of the nanocomposites at a higher CNCs content, which favored the degradation of PHB by hydrolysis. Overall, in the case of PHB/CNCs nanocomposites where TB was used as a plasticizer, the effect of CNCs on biodegradation was stronger than that of the plasticizer. However, the type of plasticizer affected the biodegradation rate. In the case of PHB/CNCs nanocomposites containing a high molecular weight plasticizer, a rather low degradation rate was noticed, the nanocomposites having a similar biodegradation behavior with PHB. Therefore, in this case, the CNCs incorporation did not lead to an acceleration of the material biodegradation, despite their hydrophilicity [101]. When the CNCs were added in a PHB/PCL blend, the biodegradability of PHB/PCL/CNCs nanocomposites tested under composting conditions was also influenced by the presence of CNCs and their concentration [115]. It was observed that an increase in the CNCs content led to an increase in the biodegradation rate of the PHB/PCL/CNCs nanocomposites as compared to the PHB/PCL blend and neat PHB. This was assigned both to the increased hydrophilicity as a result of the CNCs addition, which promoted and accelerated the hydrolytic degradation of PHB, and to the decrease in the degree of crystallinity of PHB as a result of PCL and CNCs addition [115]. 

### 5.5. Biocompatibility

**Figure 6:**
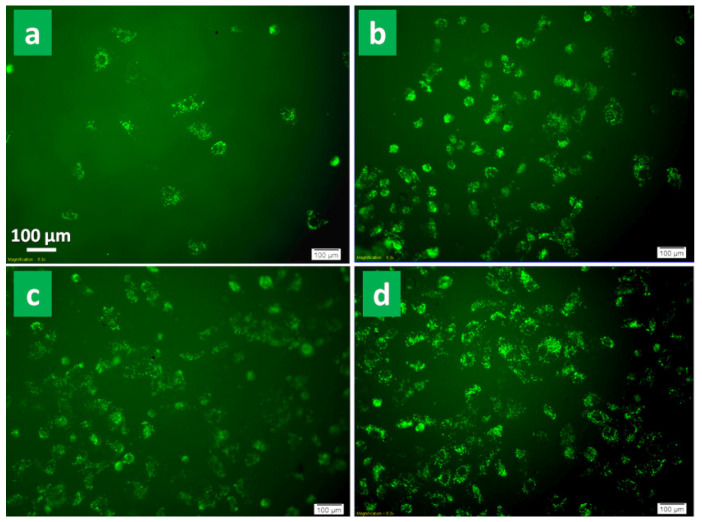
Fluorescence images showing the attachment of MG-63 on neat PHBV (**a**) and PHBV/PHCNs nanocomposites with 10% (**b**), 20% (**c**), and 30% (**d**) PHCNs. Reprinted with permission from Ref. [110]. Copyright 2014 American Chemical Society.

As combinations of two biocompatible materials, the PHB/CNCs nanocomposites may be useful for biomedical applications. Choi et al. obtained PHB/CNCs nanocomposite foams with different contents of CNCs by employing a nonsolvent-induced phase separation technique [106]. Regardless of whether THF or Diox was used as a nonsolvent, cytotoxicity tests showed that the resulted PHB/CNCs nanocomposites showed sufficiently high cell viability (more than 80%) for at least 4 days, which render them safe for potential biomedical application [106]. Furthermore, an enhanced cell adhesion was observed after CNCs addition to PHBV [110]. The cytocompatibility of the PHBV/PHCNs nanocomposites was evaluated by culturing human MG-63 cells on their surface. It was found that the addition of PHBV-grafted CNCs (PHCNs) in PHBV increased the MG-63 cells growth. Moreover, an increase in the number of living cells from the PHBV/PHCNs nanocomposites surface was observed as the content of PHCNs in the PHBV/PHCNs nanocomposites increased (Figure 6). This was explained by the higher hydrophilicity of the nanocomposites as compared to the neat PHBV which promoted cells attachment and proliferation. Therefore, the addition of PHBV-grafted CNCs improved cell−matrix interactions and the adhesion of MG-63 cell on the surface of nanocomposites [110]. 

The PHB/CNCs electrospun mats were also characterized by in vitro cytotoxicity tests using a direct contact test with L-929 cells and MTT assay [91]. The PHB/CNCs nanocomposite mats with a CNCs content of 22 wt% were proven to be noncytotoxic, supporting the viability and proliferation of the L-929 fibroblasts cells. In addition, a significant increase in the water absorption capacity of PHB, which is known to be a hydrophobic material, was observed with increasing the CNCs content in the PHB/CNCs nanocomposites mats. This was due to the presence of the numerous hydroxyl (-OH) groups on the CNCs surface, which were able to establish hydrogen bonding interactions with water molecules. Consequently, an improvement in the percentage of water absorption of around 311% was observed in the case of the PHB/22 wt% CNCs nanocomposite mats as compared to the neat PHB mats. Therefore, PHB/CNCs nanocomposites mats are more suitable as materials for scaffolds or other biomedical applications as compared to the pure PHB [91]. 

## 6. Applications of PHB/CNCs Nanocomposites

**Figure 7:**
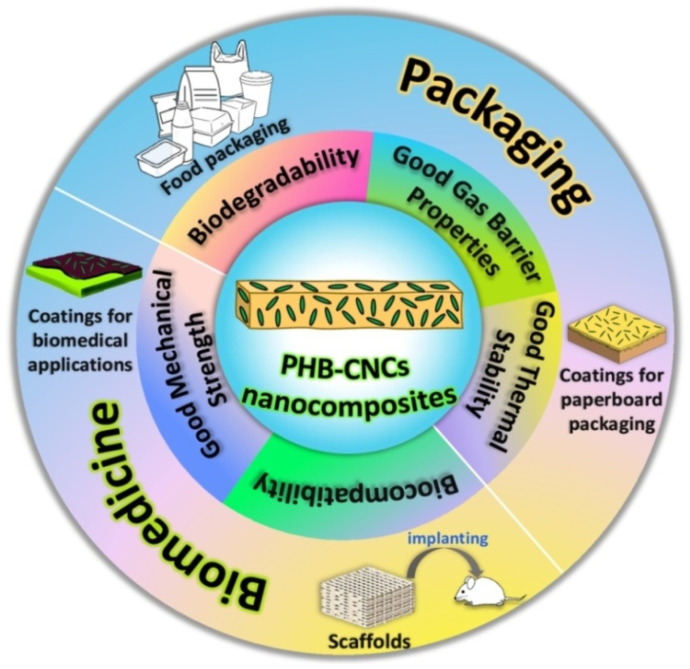
Schematic representation of PHB/CNCs nanocomposites’ applications.

Due to their biodegradability and improved thermal and mechanical features, PHB/CNCs bionanocomposites have a great market potential, especially in the medical and packaging sectors (Figure 7). In addition to the “green” character, the incorporation of CNCs derived from cheap natural resources in PHB can reduce the production costs, one of the important barriers to the widespread marketing of PHB. When intended for use as packaging materials, PHB/CNCs nanocomposites must satisfy certain ‘essentialrequirements’ like easy processing, transparency, high elongation at break and good barrier properties. Moreover, when packages reach the end of their life, the waste must ultimately decompose into mainly carbon dioxide, biomass, and water in order to have a minimal impact on the environment [123]. A major sector of food packaging is represented by the short-life packaging where biodegradability represents a compulsory characteristic. Showing a rapid biodegradation, which is in general enhanced by the addition of CNCs, PHB nanocomposites are suitable biomaterials for short-life packaging. Besides the enhanced flexibility and processability, the food packages should also show specific barrier properties. It was observed that PHB/PCL/3 wt% CNCs nanocomposites obtained through solvent casting followed by extrusion and compression molding showed balanced mechanical properties and good UV barrier properties, along with high transparency and degradation rate [115]. Therefore, the nanocomposite with 3 wt% CNCs is promising for packaging application. Luzi et al. studied the suitability of PLA/PHB/CNCs films plasticized with an oligomeric lactic acid and containing carvacrol, a natural antimicrobial additive, for food packaging [124]. The extruded films showed a good Young’s modulus and an excellent elongation at break (430%), along with a reduction of the oxygen transmission rate following CNCs incorporation. Moreover, all formulations disintegrated in less than 17 days under composting conditions, showing a weight loss higher than 80% [124]. 

Although the addition of a plasticizer is needed for a better processability and flexibility of the PHB/CNCs films intended for packaging application, their addition can negatively influence the mechanical and barrier properties. Seoane et al. studied the possibility of using PHB/CNCs nanocomposites, plasticized with tributyrin or poly (diethylene adipate), as biodegradable packaging materials [101]. The investigation of the PHB/CNCs nanocomposites’ biodegradability was performed under simulated composting conditions according to the ISO 20200 standard. The addition of small molecular weight plasticizers improved the dispersion of CNCs in the PHB matrix. Moreover, all the prepared PHB/CNC_S_ nanocomposites showed a degree of disintegration of about 90% in 21–28 days after the beginning of the disintegration tests, which, together with their promising mechanical and thermal properties recommend them, as suitable candidates for the packaging industry [101].

Besides their use as films for packaging, the PHB/CNCs nanocomposites can also serve as coatings for paperboard or other hydrophilic substrates [100]. Thus, plasticized PHB/CNCs nanocomposites were used as layers for paperboard in order to improve its mechanical properties and to protect it against moisture. The new biodegradable and low cost composite materials were characterized in terms of barrier properties and disintegration in composting conditions for the evaluation of these materials as packaging. Although reduced after the addition of CNCs, the water vapor permeability of the paperboard coated by PHB/CNCs was still higher with about one order of magnitude compared to that of the same paperboard coated by polyethylene. However, the paperboard coated by PHB/CNCs achieved a much higher disintegration level (80%) than the neat paperboard (55%) after 35 days in composting conditions, which strongly support these composites as cheap packaging materials [100].

The application of PHB and PHB/CNCs in biomedicine has a great future. For the application in the biomedical field, the PHB/CNCs nanocomposites must possess two essential characteristics, namely biocompatibility (both PHB and CNCs are inherently biocompatible) and nontoxicity. However, each biomedical application may demand supplementary characteristics such as bioactivity, biodegradability, superior mechanical strength, or a certain degree of flexibility that may require the addition of other specific components to the PHB/CNCs formulations. In contrast to PLA, PGA, or PLGA, largely used in medical applications, the biodegradation of PHB and its copolymers in living tissues does not cause medium acidification [125]. The predominant degradation product of PHB, 3-hydroxybutyric acid, is much weaker than lactic acid, the main biodegradation product of PLA and PLGA; therefore, PHB and its nanocomposites do not cause chronic tissue irritation due to a decrease of pH, which is an extremely serious problem associated with PLA implants [125]. Up to now, only in vitro studies on PHB/CNCs nanocomposites were done [91], which demonstrated their lack of toxicity. However, in vivo studies are necessary for a more rapid application of PHB/CNCs nanocomposites as scaffolds and dressings.

Besides these applications, the PHB/CNCs nanocomposites may be useful in other fields such as encapsulation of medicine or fertilizers, and even electronics and other engineering applications. 

## 7. Conclusions and Future Perspectives

The addition of CNCs in PHB is an efficient tool for designing fully biobased and biodegradable PHB/CNCs nanocomposites with improved properties. PHB/CNCs bionanocomposites proved to be suitable as replacements for fossils fuel-based materials because they have many properties comparable to that of the latter and, additionally, an insignificant carbon-footprint during processing and disposal. Even though PHB/CNCs nanocomposites have experienced a rapid advance in many fields and, especially, in packaging, there are still some scientific and technological challenges that remain open. One of them is the large improvement in interfacial adhesion and CNCs dispersion in the PHB polymer required for a strong enhancement of properties. Therefore, CNCs need to be efficiently functionalized in order to serve as effective reinforcing agents in PHB. In this respect, several approaches have been made, by using dispersing agents, compatibilizers, or grafting polymers compatible with PHB or PHBV on the CNCs’ surface. However, it should be taken into account that all the surface treatments applied to CNCs will increase the costs of the final material. Therefore, as a future outlook, there is still a need for further advancements in the treatment of CNCs and in the methods used for obtaining PHB/CNCs based nanocomposites with finely dispersed CNCs. Concerning the applications of PHB/CNCs nanocomposites, more attention needs to be paid to the specific studies such as barrier properties, migration, toxicity, and biocompatibility, especially in vivo. The deepening of such specific tests would contribute to a great extent to a clearer outline of the behavior of these materials in terms of their application. In summary, although there are some barriers that need to be overcome, recent and continuous advances in the scalability of the industrial production of PHB/CNCs nanocomposites offer great promise and potential for their utilization in the packaging and biomedical sectors, while contributing to a sustainable future and economy.

## Figures and Tables

**Table 1:** Thermal and mechanical properties of selected PHB/CNCs nanocomposites.

Polymer Matrix	CNCs Loading (wt%)	Functionalization/Additives	Processing Method	Thermal Properties				Mechanical Properties				References
				T_max_ (°C)	T_m_ (°C)	T_g_ (°C)	X_C_ (%)	Tensile Strength (MPa)	Young’s Modulus (MPa)	Elongation at Break (%)	Sorage Modulus * (MPa)	
PHBV	2	-	Melt compounding	~280	168.3	-	55.6	36.34 ± 0.50	603.91 ± 22.77	15.35 ± 0.66	-	[90]
	5			~282	168.8	-	57.6	33.28 ± 0.57	645.62 ± 22.12	11.66 ± 0.67	-	
	7			284.3	171.4	-	59.0	32.01 ± 0.50	792.98 ± 23.56	10.04 ± 0.49	-	
PHB	2	L-lactide graft polymerization/-	Melt compounding	-	153.9/166.0	-	-	-	-	-	-	[113]
PHB	2.4	-/PVAc	Masterbatch melt compounding	293.49	~160/170	-	-	20 ± 2	1100 ± 95	17 ± 1	-	[114]
	4.8			294.10	~161/170	-	-	25 ± 2	1170 ± 50	16 ± 1	-	
PHB	2.4	-/PEG		294.53	~162/171	-	-	11 ± 1	1200 ± 69	2	-	
											-	
PHBV	2.4	-/PVAc		276.20	~150/160	-	-	25 ± 1	1320 ± 192	12 ± 1	-	
	4.8			286.49	~150/160	-	-	32 ± 2	1300 ± 243	51 ± 2	-	
PHBV	2.4	-/PEG		290.77	~153/162	-	-	13 ± 1	1270 ± 127	2	-	
PHB	3	-/PCL	Solvent casting and melt compounding	257.0	161.7/168.0	-	45.7	14.5 ± 0.7	902 ± 56	4.1 ± 0.4	-	[115]
	5			251.0	151.7/160.1	-	41.0	9.3 ± 1.4	1066 ± 65	1.8 ± 0.9	-	
PHBV	2	-/PEG	Solvent casting	-	~134/151	-	-	15.5	1100	7.1	~1900	[108]
	5			-	~132/150	-	-	26.1	1760	7.8	~2500	
	2		Melt compounding	-	-	-	-	~25.2	~1700	~4	-	
	5			-	-	-	-	~24.8	~1900	~3.4	-	
PHB	2	-	Solvent casting		171.2	-	47.7	-	-	-	-	[96]
	5			-	172.0	-	57.8	-	-	-	-	
PHBV	2	butyric acid/-	Solvent casting	277	171	-	50	-	-	-	4788	[109]
	5			285	170	-	44	-	-	-	3869	
	2	lactic acid/-		282	170	-	46	-	-	-	3283	
	2	butyric acid and lactic acid/-		275	170	-	51	-	-	-	3496	
PHBV	2	-	Solvent casting	-	~132/150	~−1.7	-	~35.5	~1850	-	~1100	[97]
	4.6			-	~124/142	~−4.2	-	~19	~1790	-	~1380	
PHB	2	-	Solvent casting	~288	160.6/170.8	-	53.6	29.7 ± 3.6	2400 ± 300	1.5 ± 0.2	-	[86]
	4			~290	160.5/170.4	-	53.7	29.2 ± 1.8	2500 ± 200	1.5 ± 0.2	-	
PHB	2	-/glyceryl tributyrate (TB)	Solvent casting	~270	154.5/169.2	-	62.7	17 ± 2	1500 ± 100	1.9 ± 0.3	-	[99]
	4			~274	154.7/169.6	-	59.8	19 ± 2	1800 ± 100	1.6 ± 0.3	-	
PHB	2	-/poly[di(ethylene glycol) adipate] (A)		~287.5	155.3/169.4	−13.6	57.1	15 ± 1	1200 ± 100	2.4 ± 0.5	-	
	4			~293	154.1/168.9	−13.9	56.8	14 ± 1	1100 ± 100	2.0 ± 0.4	-	
PHB	2	-	Solvent casting	~275	~162/172	-	55.4	~24.4	~1930	~1.77	-	[98]
	4			~271	~162/172	-	55.3	~25.3	~2050	~1.8	-	
PHB	0.15	-/PEG	Solvent casting	314	160	-	-	~17.1	~185	~180		[102]
	0.45			316	162	-	-	~17.5	~80	~300		
PHBV	5	PHBV grafting/-	Solvent casting	252.6	90.5/112.2/129.6	−16.9	-	~20	~300	~6.2	-	[110]
	20			279.5	99.5/121.3/136.7	−12.1	-	~33	~43	~7	-	
PHB	5	-	Melt compounding	~308	176	-	39.6	28.1 ± 1.9	1518 ± 55	3.39 ± 0.18	1800	[116]
		-/polylactic acid (PLA) and epoxidized canola oil (eCO)	Melt compounding	~317	171	-	43.4	24 ± 1	1604 ± 66	2.93 ± 0.16	1590	
P3HB/P4HB	5	tetraethyl orthosilicate (TEOS) and methyltrimethoxysilane (MTMS) double silanization/-	Melt compounding	-	158	~−17	-	19.2	5.52	301	-	[111]
PHB	3	-	Solvent casting	290.2	170.9	-	64.2	~25.8	~1890	~4.5	-	[89]
	5			291.7	171.0	-	67.1	~22.9	~2020	~2.6	-	
PHB	5	-	Electrospinning from solution	-	183.2	-	-	4.2 ± 0.3	4931.3 ± 245.4	19.4 ± 1.7	-	[91]
	8			-	184.7	-	-	4.4 ± 0.2	5120.1 ± 215.3	18.3 ± 1.2		
PHB	2	-	Solvent casting	-	-	-	-	57.1 ± 1.2	870 ± 20	6.5 ± 1.0	-	[95]
	5			-	-	-	-	45.2 ± 1.8	1410 ± 70	3.2 ± 0.8	-	

* Storage modulus value at room temperature.

## Data Availability

Not applicable.
